# The relationship between COPD and lung cancer

**DOI:** 10.1016/j.lungcan.2015.08.017

**Published:** 2015-11

**Authors:** A.L. Durham, I.M. Adcock

**Affiliations:** Airway Disease Section, National Heart and Lung Institute, Imperial College London, Dovehouse Street, London, UK

**Keywords:** COPD, Cancer, ROS

## Abstract

Both COPD and lung cancer are major worldwide health concerns owing to cigarette smoking, and represent a huge, worldwide, preventable disease burden. Whilst the majority of smokers will not develop either COPD or lung cancer, they are closely related diseases, occurring as co-morbidities at a higher rate than if they were independently triggered by smoking.

Lung cancer and COPD may be different aspects of the same disease, with the same underlying predispositions, whether this is an underlying genetic predisposition, telomere shortening, mitochondrial dysfunction or premature aging. In the majority of smokers, the burden of smoking may be dealt with by the body’s defense mechanisms: anti-oxidants such as superoxide dismutases, anti-proteases and DNA repair mechanisms. However, in the case of both diseases these fail, leading to cancer if mutations occur or COPD if damage to the cell and proteins becomes too great.

Alternatively COPD could be a driving factor in lung cancer, by increasing oxidative stress and the resulting DNA damage, chronic exposure to pro-inflammatory cytokines, repression of the DNA repair mechanisms and increased cellular proliferation. Understanding the mechanisms that drive these processes in primary cells from patients with these diseases along with better disease models is essential for the development of new treatments.

## Highlights

• COPD is a risk factor for lung cancer beyond their shared aetiology.
• Both are driven by oxidative stress.
• Both are linked to cellular aging, senescence and telomere shortening.
• Both have been linked to genetic predisposition.
• Both show altered epigenetic regulation of gene expression.

## 1 Introduction

Tobacco smoking originated in the Mayan kingdom in 600–900 AD [1] before spreading across the globe with the advent of cross-Atlantic trade and particularly the invention of pre-rolled cigarettes in the late 19th century. Thus, smoking rates increased globally from <0.5% of the USA population from the start of the 20th century peaking in 1965 with 50% of men and 33% of women who smoked [2]. The increase in smoking correlated with an increase in the incidence of lung cancer [3].

Approximately 50% of smokers will have their lives curtailed by cigarettes: each cigarette smoked reduces life expectancy by 11 min such that males and females each lose an average of 13.2 and 14.5 years of life, respectively [2]. From 2000–2004 in the USA alone, ∼443,000 adults died due to cigarette smoking-related diseases including lung cancer (125,522), coronary heart disease (80,005) and COPD and other airway obstructive diseases (78,988) [2].

## 2 Lung cancer

The lifetime risk of developing lung cancer is 17.2% for males and 11.6% for females in smokers compared with 1.3% and 1.4% respectively for non-smokers [4]. Lung cancer is the leading cause of cancer-related death worldwide [5], [6] with only a 16% 5-year survival rate [7], The number of lung cancer deaths is expected to rise to ten million deaths per year by 2030 [5].

Lung cancer is caused by mutations in oncogenes leading to the proliferation of the mutated cells and the formation of a tumor. Additional mutations can further transform the benign tumor to an invasive cancer, a process marked by metastatis (spread), invasiveness and anaplasia (loss of cell type specific features) [8]. Lung cancers usually originate from the basal epithelial cells and are classified into two types, non-small cell lung cancer (NSCLC) and small cell lung cancer (SCLC). NSCLC accounts for approximately 85% of lung cancer cases [9] with adenocarcinoma accounting for 40% of the total and large cell carcinoma for 5–10%. The remaining NSCLC (30%) derive from squamous cells. The other cancers are termed small cell lung carcinomas (SCLC), which are composed of smaller than normal, undifferentiated cells [8].

## 3 COPD

COPD is a progressive and ultimately fatal deterioration of lung function over time [10]. COPD has a marked effect on a patient’s quality of life affecting up to 50% of smokers [11]. COPD was the third most common cause of death worldwide in 2010 [12] and ranked fifth worldwide in terms of burden of disease [10].

Damage to the lungs in COPD is caused by oxidative stress (both exogenous from smoking and endogenous), inflammatory cytokine release, protease activity (due to the protease: anti-protease imbalance) and autoantibody expression [13]. These in turn can lead to airway destruction, air trapping and lung hyperinflation.

## 4 COPD and lung cancer are linked diseases

COPD and lung cancer are caused by cigarette smoking and there is increasing evidence linking the two diseases beyond a common etiology. COPD is an independent risk factor for lung carcinoma, particularly for squamous cell carcinoma [14] and lung cancer is up to five times more likely to occur in smokers with airflow obstruction than those with normal lung function [15]. Even excluding factors such as over diagnosis COPD patients still have twice the risk of lung cancer development [16]. The high prevalence of lung cancer in COPD suggests that there may be common mechanisms, such as premature aging in the lungs, genetic predispositions to either disease or common pathogenic factors, such as growth factors, activation of intracellular pathways or epigenetics. [17].

## 5 Lung cancer and COPD: Diseases of the aging lung

The probability of developing cancer increases with age [18] and the median age of onset for lung cancer is 66 years old [19]. COPD principally affects smokers aged over 40 and is 2.5 times higher in over 60 year olds [11]. The normal decline in lung function with ageing is accelerated in patients with COPD leading to premature loss of lung function [20], [21]. Aging is principally driven by failure of organs to repair DNA damage by oxidative stress (non-programmed aging) and from telomere shortening as a result of repeated cell division (programmed aging). These defects are both present in COPD [22].

## 6 Oxidative stress is a causative agent of both diseases

Cigarettes contain approximately 10^15^ free radicals per puff [23], [24], including reactive nitrogen and oxygen species (RNOS) [25]. In addition to exogenous RNOS, mitochondrial respiration is a major source of RNOS generation and mitochondrial dysfunction is present in many cancers [26]. RNOS damage cells through a number of mechanisms including DNA damage (especially mitochondrial DNA) lipid peroxidation, oxidation of amino acids and oxidation of inorganic enzyme co-factors.

### 6.1 Oxidative stress damages DNA

**Fig. 1:**
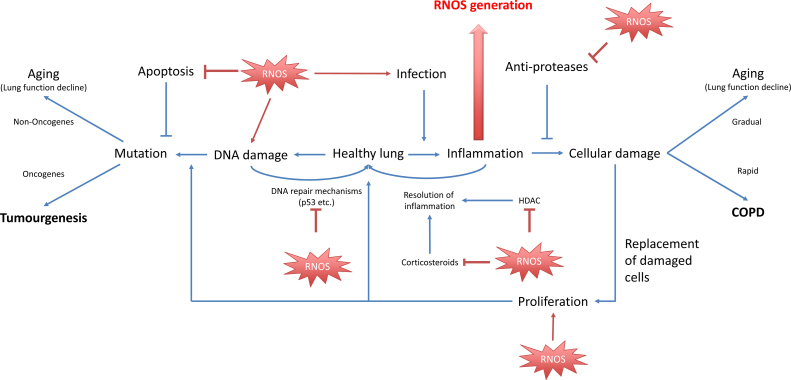
Reactive oxygen and nitrogen species (RNOS) derived from both exogenous and endogenous sources drive many of the pathways in both COPD and lung cancer. RNOS can react with DNA, leading to DNA damage, which if not correctly repaired leads to mutations. Mechanisms that prevent mutation, including DNA repair and apoptosis can be inhibited by RNOS activity. Additionally RNOS can contribute to susceptibility to infection and drive inflammation in the lungs. Inflammation can lead to further cellular and DNA damage, both through the generation of further RNOS and also through the action of cytokines and proteases. RNOS are capable of inhibiting the protective mechanisms, such as anti-proteases. Damage to the lungs is repaired by processes including cellular proliferation, which can in turn promote tumourgenesis.

The free radical hypothesis of aging proposes that RNOS drives the accumulation of cell and DNA damage [27] and elevated levels of oxidative stress are seen in many cancers [22], [28], [29], [30], [31], [32], [33]. Oxidative stress drives cancer initiation through DNA damage: point mutations, single stand breaks (SSBs) and double strand breaks (DSBs) and DNA cross-linking [28], [34], which if incorrectly repaired, results in mutations. The number of somatic mutations, which lead to cancer, accumulates with age in part due to continuous RNOS exposure. Hydroxyl radicals and peroxinitrate are especially implicated in DNA damage [33] (Fig. 1). RNOS can lead to the degradation of proteins, including tumor suppressors leading to cell division and decreasing apoptosis and DNA repair [33].

RNOS also plays a role in cancer promotion and progression [35]. RNOS is an intracellular signal and activates proliferative and inflammatory pathways, including the c-Jun N-terminal kinases (JNK) dependent mitogen activate protein kinase (MAPK) pathway which, in turn, drive proliferation [36]. Growth factor signal transduction via RNOS is associated with oxidation of thiol groups of tyrosine phosphatases, which results in their reversible inactivation and facilitates proliferation [36]. Additionally NO can increase angiogenesis through vascular endothelial growth factor (VEGF) stimulation and increases tumor growth and cell invasion properties [37].

### 6.2 Oxidative stress causes inflammation

RNOS also stimulates the production of inflammatory mediators either directly [38] or indirectly [39]. Cells directly detect RNOS via the ROS receptor/proto-oncogene ROS1 [40]. ROS1 activates the phosphoinositide 3-kinase (PI3K)-mTOR signaling pathway [41] and other proteins related to cell differentiation, proliferation, growth and survival including AKT1, MAPK1, MAPK3, IRS1 and PLCG2. Furthermore, ROS activates NF-κB thereby upregulating the expression of numerous immune and inflammatory genes [42].

RNOS can alter protein structure and function by modifying amino acid residues, inducing protein dimerization, and interacting with Fe-S moieties or other metal complexes. In COPD these post-translational mechanisms include the nitration of histone deacetylase (HDAC) 2, leading to its inactivation and degradation resulting in the prolonged inflammatory phenotype seen in patients [43], [44]. Additionally, RNOS can modify various proteins, rendering them auto-antigenic (i.e., immunoinflammatory) [45]. (Summarized in Fig. 1). Therefore oxidative stress can be a key cause of both proliferation (lung cancer) and inflammation (COPD) in the lungs.

## 7 Telomere shortening is a risk factor in COPD and lung cancer

Telomeres are repetitive nucleotide sequences located at the ends of the chromosomes which protect against the progressive shortening that occurs with DNA replication. Telomere shortening is associated with cell aging and senescence; with cells unable to divide once their telomere shorten beyond a crucial point—termed the Hayflick limit [46]. Once cells reach senescence the telomere nucleoprotein loses structural integrity and cell division is arrested in a process involving the tumor suppressor proteins p53 and Retinobastoma protein (Rb), which regulate cell cycle progression [46]. Entry of the cell into replicative senescence has been suggested as a tumor suppressing mechanism, preventing replication of cells most likely to form neoplasms.

Some cells, however, are able to bypass senescence through inactivation of the Rb and p53 signaling pathways, enter ‘crisis’ and continue to replicate [46]. Telomeres are critically short in these cells and no longer protect the chromosome ends. The crisis phase is characterized by genetic instability and widespread cell death by apoptosis [46]. An immortalized clone of the cell (1 in 10^7^ cells) can emerge, which maintains telomere length through cell divisions. Both COPD and lung cancer are associated with shortened telomere length, and importantly telomere shortening can be driven by cigarette smoking [47].

Several studies have shown a link between short telomere length and the development of lung cancer [48], [49], [50], [51] and poor prognosis [51]. Similarly there is evidence that shorter telomeres are associated with COPD [52] and short telomere length may contribute to inflammation in COPD [53]. Studies using murine models have also shown that shortened telomeres increases susceptibility to emphysema [54]. Since telomere shortening is accelerated by smoking, cells may reach their Hayflick limit more rapidly and enter replicative senescence causing COPD, or enter crisis where the accumulation of mutations leads to an immortalized cancer clone cell.

## 8 Genetic predisposition to lung cancer and COPD

There is evidence for familial susceptibility in both COPD and lung cancer. This familial susceptibility appears to be linked and not just associated with the common consumption of cigarettes; implying that the underlying genetic predisposition to both diseases may be the same or reflect the link between the immune system, inflammation and cancer [55]. Linkage studies have implicated regions in chromosome 6 as being linked to both diseases [55], [56], [57]. Furthermore, GWAS studies in large COPD and lung cancer cohorts have found the same risk loci including *CHRNA3* and *CHRNA5* SNPs (15q) and regions at 4q31 (*HHIP*), 4q24 (*FAM13A*) and 5q (*HTR4*). Nicotine addiction may explain the overlap in risk loci between lung cancer, smoking behavior and COPD [58]. Epithelial to mesenchymal transition (EMT) and inflammation are pathogenic features of COPD and lung cancer and the rs7326277TT genotype in *VEGFR1*, which promotes inflammation, EMT and tumor growth, is a susceptible locus for both COPD and lung cancer [59]. Several studies have demonstrated that polymorphisms in the anti-inflammatory gene *IL10* are associated with increased rates of lung and other cancer [25], [60].

## 9 Epigenetics in lung cancer and COPD

In addition to genetic changes, epigenetic changes including DNA methylation, covalent histone modifications, microRNAs (miRNAs) expression and nucleosome remodeling have also been reported to play an important role in the development of cancer [61] and COPD [62]. DNA methylation, which is a reversible modification of DNA structure, adding a methyl group to the 5′ position of a cytosine residue, often as part of a CpG island or cluster [63], of tumor suppressor genes including APC, CDKN2, BRCA1, Rb and MDM2 contributes to increased proliferation [64], [65]. Hypermethylation of tumor suppressor and other gene promoters is observed in the majority of lung cancers [66]. The reversible nature of promoter hypermethylation makes it an attractive target for cancer therapy.

Unsupervised hierarchical clustering of DNA methylation patterns results in 3 lung cancer clusters [67]. Lung adenocarcinomas in Cluster 1 developed from an inflammatory background in COPD in heavy smokers and were locally invasive. Overall, DNA methylation profiles may reflect carcinogenetic factors such as smoking and COPD and may distinguish patients with distinct types of NSCLC.

Recently an epigenome wide association study (EWAS) has been carried out to examine links between gene methylation in COPD and lung cancer [68]. This study identified that DNA methylation and repression of 2 genes, *CCDC37* and *MAP1B*, was significantly associated with both COPD and lung cancer [68]. Furthermore, COPD patients had a higher level of methylation and gene repression than non-COPD patients with the greatest degree of methylation seen in cancer patients with COPD.

Although alterations to DNA methylation have been examined in COPD these are mostly linked to hypomethylation of immune-modulatory genes [69] or in the SERPINA1 gene coding for alpha1-antitrypsin [62] and linked to gene overexpression.

DNA coiling around histones is a dynamic process controlled by histone acetylation and methylation and alterations to the activity to the enzymes which control this process can have a large impact on gene expression. Histones are acetylated by histone acetyl transferase proteins (HAT), which unwinds the DNA allowing transcription: acetyl groups are removed by HDACs resulting in gene silencing. Cigarette smoke reduces the expression and activity of HDAC2 both at the protein and mRNA level [70] and HDAC2 plays a key role in inflammation in COPD [71]. In contrast to COPD, where therapeutic attempts to restore HDAC activity have been trialed [72], HDAC inhibitors have been tested in lung cancer [73]. This may reflect the relative importance of deacetylation of proteins including p53, c-Myc, NF-κB, HIF-1α, HSP90 [73] or that HDAC inhibitors and HAT mimics may produce similar cellular functions due to non-selective histone acetylation preventing selective transcription factor DNA binding [74].

Non-coding RNAs include microRNAs (miRNA), which are small non-coding, single stranded RNA molecules, of 19–25 nucleotides in length [75]. MiRNAs are capable of binding to full length mRNA sequences and alter their translation into protein. The effect of inducing or repressing microRNA expression can influence most biological processes, including cell fate specification, cell proliferation, DNA repair, DNA methylation and apoptosis and provide pro-inflammatory or anti-inflammatory stimuli. Importantly, miRNAs play an essential role in the development of both the adaptive and innate immune system [76].

The interactions of miRNAs and mRNAs and their role in disease is not yet fully understood, but may be potential drivers and biomarkers of disease [77]. Several miRNAs are linked to both inflammation and proliferation. For example *miR-1* has been linked to cigarette smoking-related conditions such as heart disease and cancer [75] and is down-regulated in skeletal muscle of patients with COPD compared with non-smoking controls and expression correlated with clinical features [78]. *miR-21* has been shown to play a role in both inflammation and cancer [25]. Another miRNA *miR-146a* has been shown to downregulate both inflammation and cancer cell proliferation [79].

## 10 COPD as a driver of lung cancer

COPD and lung cancer share many common pathways for activation, and inflammation and cancer are closely linked. As almost all cancerous tissues show inflammation and a number of inflammatory diseases can predispose to cancer [25], [80], it is possible that that the chronic inflammation in COPD is a potent driver of lung cancer development as evidenced by the efficacy of non-steroidal anti-inflammatories as anti-cancer treatments [25]. Inflammation is a key source of RNOS [33] and RNOS levels are persistently high in COPD.

Mitochondria are the major cellular source of RNOS [26] and the mitochondrial dysfunction seen in COPD [81] may link COPD with the development of lung cancer. Lung endothelial cell apoptosis is regulated by the mitochondrial transcription factor A (mtTFA). The gene for mtTFA is methylated in COPD patients with squamous cell lung cancer leading to reduced expression and loss of normal mitochondrial function resulting in endothelial cell apoptosis [82].

Inflammatory mediators can influence the cancer micro-environment, and the expression of cytokines is vital to drive the immune response to prevent cancer formation. For example IFNγ knockout mice are more susceptible to carcinogens than wild-type mice [83]. However inflammation can also drive carcinogenesis. Aberrant cytokine signaling in chronic inflammation can drive cell growth differentiation and apoptosis [83]. Numerous cytokines are associated with cancer development, for example macrophage colony stimulating factor (M-CSF) has been linked to breast cancer spread in mice models [84].

Increased IL-17 expression is associated with the severity of COPD [85] and promotes chronic inflammation. In a murine model of lung cancer, lack of IL-17A, but not IL-17F, reduced tumor cell proliferation and inflammatory mediator expression [86]. This data highlights a possible novel approach to the treatment of COPD-associated lung cancer.

Chronic inflammation is associated with the overexpression of the transcription factor NF-κB, which is a key mediator of inflammation-induced carcinogenesis [87]. NF-κB induces the expression of many pro-inflammatory cytokines such as *IL1*, *IL6*, *IL8* and *TNFα* as well that of key components of the cell cycle including the cyclins D1, D2, D3, E1 and various cyclin dependent kinases (CDKs) [87]. Additionally NF-κB can contribute to cancinogenesis by suppression of p53, by upregulating the levels of p53 E3 ligase, and thereby reducing p53 stability. Other cellular pathways which are involved in carcinogenesis also show cross-talk with inflammatory pathways. The PI3K pathway is an important driver of proliferation and the suppression of cell apoptosis and is activated in COPD [88]. Aberrant expression of growth factors is linked to tissue remodelling in response to cigarette smoke in COPD patients and plays a role in lung cancer. For example, levels of EGF receptors are higher in COPD patients [89] and in lung cancer [90].

Another common pathway to inflammation and proliferation is the Wnt pathway. Canonically the Wnt proteins are extracellular messenger proteins that bind to the disheveled (Dsh) receptor on the cells; this in turn leads to a signaling cascade that ultimately leads to the buildup of the transcription factor β-catenin. Activation of Wnt proteins and β-catenin is increased in COPD patients [91] and may be linked to premature aging in the lungs [92]. As drivers of development and cellular proliferation aberrant Wnt pathway activation is an important driver of many cancers and activation of Wnt/Beta catenin pathway is associated with faster lung cancer development in mice [93]. However, whilst strongly linked to other cancers, e.g., colon cancer [94], mutations in Wnt/APC are not strongly associated with lung cancer.

The chronic inflammation in COPD also causes lung damage which results in cell division in an effort to restore homeostasis. The increased rate of cell division, especially if paired with increased DNA damage due to smoking [22], greatly increases the probability mutations thereby increasing the chance of carcinogenesis. The repair processes in the lung include epithelial-mesenchymal transition (EMT), in which epithelial cells transform to mesenchymal cells in order to translocate to the site of damage, where they revert to epithelial cells. EMT is driven by transforming growth factor (TGF) and is a process has been linked to both COPD [95] and lung cancer [96].

The physiological conditions within the lung caused by COPD, rather than the chronic inflammation underlying the disease *per se*, may also contribute to the development of lung cancer. The lungs of COPD patients are hypoxic due to air trapping and reduced air flow. Hypoxic conditions stimulate the activation of the transcription factor hypoxia inducible factor (HIF) 1-alpha. HIF-1α induction is also seen in cancer cells due to the local hypoxic environment. HIF-1α regulates over 200 genes and activates glycolysis, immortalization through telomerase activation, stoppage of differentiation and can inhibit apoptosis [36]. Recent comparisons of lipidomic profiles in sputum from COPD patients [97] suggest that COPD leads to changes in lipid profiles including increased ceramide levels. These changes in lipid metabolism in turn, may alter other physiological responses, including the hypoxia response and EGFR signaling and may play a role in the link between COPD and cancer [97].

## 11 Therapeutic implications

Lung cancer remains one of the most fatal forms of cancer, both due to lack of diagnosis and lack of effective treatments. Screening of COPD patients for the development of cancer, for example through CT scans, has been suggested as a potential method enable early detection and thereby to improve outcomes. However, this approach of increased surveillance is hampered by the lack of sensitivity of treatment and the large numbers of false positive diagnoses that result [16]. Potentially with improved understanding of the links between the diseases more selective biomarkers will become available making this approach viable.

It is increasingly recognized that, due to the range of drivers of cancer, personalized medicine approaches based on the individual’s cancer are important. For example patients can now be tested for alterations in Epidermal growth factor receptor (EGFR) or vascular endothelial growth factor (VEGF) and, when appropriate treated with specifically targeted drugs for example EGFR inhibitor Erlotinib [98] or, less successfully using VEGF binding monoclonal antibodies, such as Ramucirumab [99]. The efficacy of these drugs depends upon the presence of receptor mutations and just as importantly it is likely that specific subphenotypes of COPD will be susceptible to anti-inflammatory and/or antioxidant therapies that will impact upon the incidence of lung cancer. The combination of selective biomarkers in carefully stratified at-risk patients will be necessary to achieve optimal therapeutic effects.

It may therefore be possible to, in future, treat COPD patients with specifically targeted therapies to reduce the risk of the patients developing lung cancer. For example, owing to the important role of oxidative stress in both diseases it has been suggested that anti-oxidant therapy, for example vitamin C, vitamin E or N-acetyl cysteine (NAC) [100], may be of benefit to patients, both reducing exacerbations and inflammation and also reducing lung cancer incidence. However, the results from trials of NAC treatment in COPD are mixed often failing to improve quality of life or reduce exacerbations due to inadequate dosing [101]. Furthermore, recent work in mice indicates that treatment with antioxidants was associated with an increased cancer risk in a COPD model [102].

The potential problems with the use of antioxidants to prevent the development of lung cancer in all at risk patients highlights the heterogeneity of both diseases and therefore the need to develop specific, targeted treatments for the most at risk sub-populations of COPD patients to prevent them from developing lung cancer. In order for such an approach to be feasible however we will need to increase our knowledge of both diseases and their links and identify biomarkers to both improve screening for the most at risk patients and develop effective treatments.

## 12 Conclusions

Whilst the exact mechanism(s) underlying the increased incidence of lung cancer in patients with COPD is currently unknown, the two diseases are closely linked at a molecular level. Further research to elucidate the relationship between these two diseases may provide not only insights into the their development but may also create the possibility of cross-over treatments being developed, whereby anti-inflammatories developed for COPD may be beneficial in lung cancer and anti-cancer drugs may play a role in the future treatment of COPD.

## Conflicts of interest

ALD has no conflicts of interest. IMA has received consultancies, honoraria, and travel and research grants from GSK, AstraZeneca, Johnson & Johnson, Chiesi, Pfizer, Boehringer Ingelheim, Novartis and Vectura.
